# Dynamic Coding for Cognitive Control in Prefrontal Cortex

**DOI:** 10.1016/j.neuron.2013.01.039

**Published:** 2013-04-24

**Authors:** Mark G. Stokes, Makoto Kusunoki, Natasha Sigala, Hamed Nili, David Gaffan, John Duncan

**Affiliations:** 1 Oxford Centre for Human Brain Activity, University of Oxford, Oxford OX3 7JX, UK; 2 Department of Experimental Psychology, University of Oxford, Oxford OX1 3UD, UK; 3 MRC Cognition and Brain Sciences Unit, University of Cambridge, Cambridge CB27EF, UK; 4 Brighton and Sussex Medical School & Sackler Centre for Consciousness Science, University of Sussex, Sussex BN1 9RR, UK

## Abstract

Cognitive flexibility is fundamental to adaptive intelligent behavior. Prefrontal cortex has long been associated with flexible cognitive function, but the neurophysiological principles that enable prefrontal cells to adapt their response properties according to context-dependent rules remain poorly understood. Here, we use time-resolved population-level neural pattern analyses to explore how context is encoded and maintained in primate prefrontal cortex and used in flexible decision making. We show that an instruction cue triggers a rapid series of state transitions before settling into a stable low-activity state. The postcue state is differentially tuned according to the current task-relevant rule. During decision making, the response to a choice stimulus is characterized by an initial stimulus-specific population response but evolves to different final decision-related states depending on the current rule. These results demonstrate how neural tuning profiles in prefrontal cortex adapt to accommodate changes in behavioral context. Highly flexible tuning could be mediated via short-term synaptic plasticity.

## Highlights

► Task instruction triggers a dynamic trajectory in a population of prefrontal neurons ► Task rules are maintained in a stable low-activity state that differentiates context ► The prefrontal network is temporarily tuned by the cue for flexible decision making ► Population dynamics are flexibly routed through state space for adaptive behavior

## Teaser

Stokes et al. use dynamic pattern analysis to characterize how prefrontal cortex establishes, maintains, and uses flexible neural states for task-dependent behavior. Changes in decision rule trigger a dynamic population response that temporarily configures the network for flexible decision making.

## Introduction

The brain must constantly adapt to accommodate an enormous range of possible scenarios. In a complex dynamic environment, the behavioral relevance and/or meaning of sensory input critically depends on context. Therefore, changes in behavioral context demand a shift in the way information is processed. Here, we explore how coding in prefrontal cortex (PFC) rapidly shifts between specific processing rules according to experimentally manipulated context.

Prefrontal cortex has long been associated with flexible cognitive function. Damage to PFC is classically associated with reduced cognitive flexibility in both humans (Luria, 1966) and nonhuman primates (Rossi et al., 2007). Similarly, in studies using fMRI, lateral PFC is typically more active when participants perform tasks that demand cognitive flexibility (Wager et al., 2004). Numerous influential theories propose a key role for PFC in representing task-relevant content and rules in a temporary working memory (WM) store for guiding flexible behavior (Baddeley, 2003; Miller, 2000; Miller and Cohen, 2001).

Neurophysiological recordings suggest that PFC is capable of maintaining task-relevant information in a durable distractor-resistant WM format (Miller et al., 1996) that reflects future behavioral goals (Rainer et al., 1999). Previous studies have also suggested that activity in single prefrontal cells reflects the current task rules (White and Wise, 1999), such as a variable stimulus-response mapping (Wallis et al., 2001). However, it remains unclear exactly how activity states representing such important task parameters can be used to guide subsequent decision making and action. An adaptive coding model proposes that context-specific task parameters directly shape the tuning profile of PFC (Duncan, 2001; Duncan and Miller, 2002). Prefrontal neurons are not inherently tuned to specific features in the world, but rather adapt their tuning profiles to represent input according to task relevance. Within this framework, changing task parameters shift the response properties of the network, altering the way stimuli are coded and behavior produced.

Classification learning tasks demonstrate the basic principles of adaptive coding in PFC (Cromer et al., 2011; Freedman et al., 2001; Li et al., 2007; Roy et al., 2010). After monkeys have been trained to classify novel stimuli according to an arbitrarily defined category boundary, individual neurons in PFC display tuning profiles that are aligned with the task-relevant decision space (Freedman et al., 2001). Multivariate pattern analyses of the same data confirm task-dependent coding at the neural population level (Meyers et al., 2008). Similar shifts in tuning have been observed in human PFC using pattern analytic methods to infer the representational nature of the population response measured with fMRI (Li et al., 2007). In some cases, extensive training could establish novel tuning profiles in PFC via mechanisms of long-term synaptic plasticity. However, analogous tuning shifts can also be observed without extensive training in human PFC (Woolgar et al., 2011) and in monkey PFC, despite trial-by-trial shifts in decision rules (Roy et al., 2010; Watanabe, 1986). A rapid mechanism for adaptive coding in PFC is necessary for implementing such flexible shifts in context-dependent tuning.

In this study, we explore trial-by-trial shifts in coding within monkey PFC using a delayed paired-associate task. An instruction cue at the start of each trial controls how subsequent choice stimuli should be categorized as behavioral targets or nontargets. Time-resolved pattern analysis of a population of neurons in PFC reveals a dynamic trajectory through multidimensional state space triggered by the instruction cue. Population-level activity then settles into a low-activity state during the memory delay. Although behavioral context (classification rule) can be decoded during this delay period, the discriminating pattern is orthogonal to the neural patterns that discriminate either cue or target stimuli at the time of presentation. These results suggest that the stable activation state observed during maintenance reflects the temporarily configured network state in PFC that is dynamically tuned to respond to input according to the current task goals.

The response to choice stimuli further confirms that the cue-configured network state is adaptively tuned to map stimuli to the appropriate behavioral decision based on task context. The temporarily tuned prefrontal network rapidly transforms the coding space from differentiating the physical properties of choice stimuli to settle into a state that clearly represents the context-dependent behavioral choice. We suggest that cue processing could trigger a temporary but systematic shift in synaptic efficacies within a network of prefrontal cells (Zucker and Regehr, 2002). This distinct neurophysiological state could then shape a trajectory through state space that effectively maps distinct stimuli to the appropriate decision value according to context (Jun et al., 2010; Machens et al., 2005).

## Results

**Figure 1:**
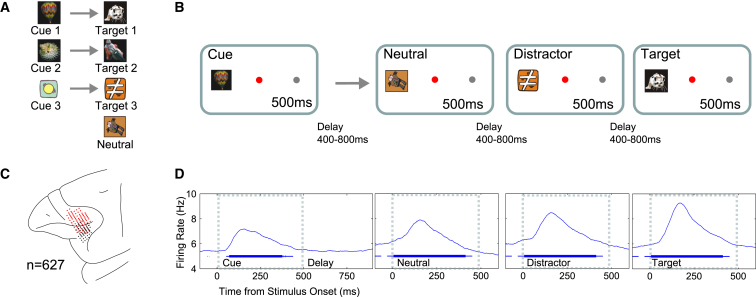
Task, Recordings, and Overall Activity Profile (A) Arbitrary associations between cue and target stimuli were learned prior to the recordings reported here. (B) The cue stimulus at trial onset determined the current target. There followed a series of 0–3 nontargets, followed by the cued target, all in the same location (right or left of fixation, randomly varying across trials). The animal was required to maintain central fixation until offset of the target and was then rewarded for a saccade to the stimulus location. Each nontarget was randomly either a neutral stimulus (a stimulus never used as a target (see A) or a stimulus paired with a different cue and hence serving as a target on other trials (here termed distractor). The presentation order in the task schematic is illustrative only. Actual presentation was randomized but always ended with the target. (C) Schematic diagram of recording sites, illustrated by red and blue symbols for monkeys A and B, respectively. Recording sites for monkey A (right hemisphere) have been transferred to the left. (D) Mean firing rate of the recorded population during each epoch of the task, as a function of time from each stimulus onset. The duration of stimulus presentation is indicated by the gray rectangle. Significantly elevated firing rate relative to 100 ms pretrial baseline is indicated by the thin (p < 0.05) and thick (p < 0.0001) blue line.

As described in more detail previously (Kusunoki et al., 2009, 2010; Sigala et al., 2008), monkeys were first trained to associate three cue stimuli to three choice stimuli (Figure 1A). Neurophysiological data were then collected in a delayed paired-associate recognition task, with a cue at the onset of each trial indicating the current target (see task structure in Figure 1B). Data were recorded from a sample of 627 randomly selected neurons in lateral PFC (Figure 1C). Unless otherwise stated, data were averaged across visual hemifields and smoothed with a 50 ms sliding average. The mean activity profile for the population of prefrontal neurons is shown in Figure 1D as a function of time and stimulus type (cue and types of choice stimuli: neutral, distractor, and target; for definitions see Figure 1, legend). Each stimulus increased overall network activity, peaking around 150–200 ms and largely returning to baseline by stimulus offset. The data suggest that peak response was higher for distractor relative to neutral stimuli and maximal for the target.

In this task, trial types 1 to 3 were defined by the cue at trial onset, indicating which stimulus was currently the target. The task required that trial type information be maintained throughout each delay to enable correct classification of the next choice stimulus. Similarly, the decision for each choice stimulus was to be maintained until stimulus offset, when the “go” versus “no-go” response could be made (see Figure 1, legend). Despite these maintenance demands, the activity of the PFC population as a whole was characterized by bursts of activity at the onset of each stimulus, followed by return to a net low-activity state between each stimulus and the next.

### Cue Processing: Population Dynamics

**Figure 2:**
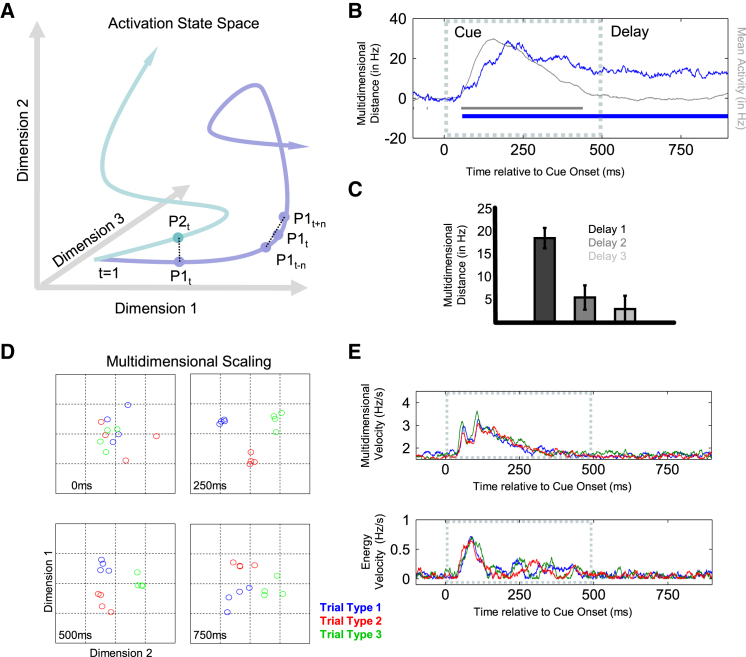
Neural Population Dynamics (A) Schematic of two different trajectories through a three-dimensional state space. The distance between two condition-specific states at time *t* reflects the multidimensional distance in the population response: d(P1_t_, P2_t_). Within-condition distance between earlier and later states represents change in position as a function of time, i.e., velocity: d(P1_t-*n*_, P1_t+*n*_)/2*n*. (B) The mean multidimensional distance between the three trial types is shown in blue as a function of time, and the significant periods of above-chance discrimination are indicated by the significance bar along the x axis (p < 0.05, cluster-based correction of multiple comparisons, see Experimental Procedures). For reference, the overall mean activity level (network energy) is shown in gray (right axis) and gray the significance bar indicates above-baseline activity (p < 0.05). (C) Bar plot of the multidimensional distance between trial types calculated for the first delay period in a trial (delay preceding first choice stimulus), a second delay period (present only when first choice stimulus was a nontarget), and a third delay period (present only when first two choice stimuli were nontargets). Error bars represent 95% confidence intervals. (D) Multidimensional distance between trial types is visualized using multidimensional scaling (MDS). Data points are four independent estimates of the population response associated with each trial type, plotted as a function of the first two dimensions at 0 ms, 250 ms, 500 ms, and 750 ms from cue onset. The complete time course of trial type-specific clustering in state space is available as Movie S1. (E) The top panel plots the estimated instantaneous velocity through multidimensional state space for each trial type, as a function of time. The bottom panel shows the equivalent velocity plot for overall energy change.

The evolution of neural processing can be traced through multidimensional space, where the activity state is an n-dimensional coordinate representing the instantaneous firing rate of *n* neurons at time t (Figure 2A). The coding trajectory is the path linking the sequence of activation states at each time point, and the multidimensional distance between positions in state space for specific conditions reflects the difference in the overall population response. Euclidean distance is always positive and scales with activity level; therefore, we subtract the median of the null distribution (estimated via randomized permutation testing, see Experimental Procedures) from the multidimensional distance measures.

A significant difference between trial types emerges as early as 50 ms after the presentation of the cue stimulus (Figure 2B). The trajectories continue to diverge until reaching a peak distance at around 230 ms before reducing to a lower plateau in the delay period. For comparison, we also replot in gray mean activity across the whole cell sample to show the overall energy of the activation state. The cue-related separation of population trajectories coincides with increasing energy level, but differentiation also persists even after the overall activity has returned to baseline in the delay period. These results show that, in the delay period, the network has settled back into a low-energy state that nevertheless differentiates between the context conditions. For completeness, we also estimated the multidimensional distance between trial types during the second and third delay periods within a trial (Figure 2C). The bar plot shows a reduction in pattern differentiation after each stimulus. A similar reduction in trial type discrimination was previously described using univariate statistical approaches (Kusunoki et al., 2009), though here we additionally find evidence that significant trial type discrimination persists into the second delay period (p < 10^−3^). There was also a trend for above-chance trial type discrimination in the third delay period (p = 0.056).

The evolution of trial type discrimination in the prefrontal network was visualized using multidimensional scaling (MDS, see Experimental Procedures). Four independent estimates of population activity for each trial type (color coded) are plotted against the first two dimensions (see Figure 2D), revealing a clear transition toward a state space that differentiates activity associated with the three trial types. In line with the above analyses, clustering becomes somewhat weaker, though still clearly visible, with offset of the cue and entry into the subsequent delay (the full time course is provided in the Movie S1).

The speed with which the response trajectory travels through state space is calculated from the average rate of change in state space within each condition as a function of time (d(P1_t-n_, P1_t+n_)/2n in Figure 2A). There was an initial rapid acceleration at around 40 ms (Figure 2E, top), which peaks in velocity at around 60 ms. This early peak is followed by a subsequent velocity dip at 85 ms, before accelerating again to a peak at around 110 ms. The first peak approximately corresponds to the earliest separation of cue-related trajectories observed in the distance metric (Figure 2B), whereas the second velocity peak approximately corresponds to the rapid increase in the separation between cue conditions beginning at 100 ms. Similarly to the overall firing rate, velocity of the cue-related trajectories also returns to prestimulus baseline levels by 400 ms. This suggests that the low-energy state in the delay period is also stable across time.

Importantly, this velocity metric is sensitive to changes in the state of the network, even if the overall energy of the system remains constant. Therefore, multidimensional velocity provides a richer measure of the population dynamics than overall change in activity levels (shown in Figure 2E, bottom), which reveals only a single dominant peak at around 85 ms corresponding to the initial increase in firing at stimulus onset, followed by a second smaller increase in energy change at around 250–300 ms that tracks the gradual decrease in firing rate observed across the population.

Overall, these initial analyses show that the transient increase in neural firing triggered by the instruction cue is associated with a rapid configuration of activity in state space that differentiates trial type. Activity then settles into a relatively low-energy stable state toward the offset of the cue and into the delay period. Although separation by trial type becomes less distinct during this more quiescent phase, the population response remains statistically separable.

### Dynamic Population Coding of Trial Type: Time Specificity versus Time Stability

**Figure 3:**
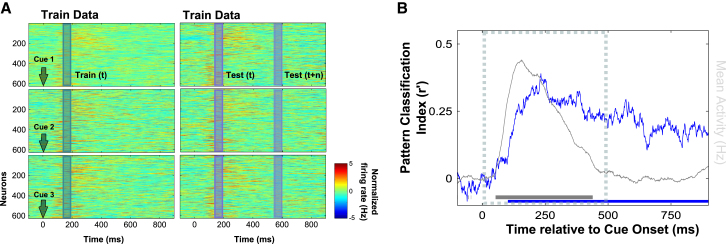
Pattern Classification (A) The pattern classifier is trained to discriminate trial types based on the population response observed within a 50 ms time window and validated using test data at the same equivalent time window (within-time classification) or at a different time window (cross-temporal classification; see Figure 4). (B) Mean within-time classification index between trial types is shown in blue as a function of time. For reference, the overall population mean firing rate is shown in gray (right axis). The blue bar along the x axis indicates periods of above-chance classification, and gray significance bar indicates above-baseline activity (p < 0.05).

To explore the dynamic evolution of activity states discriminating different trial types, we exploited a cross-temporal variant of pattern classification (see schematic in Figure 3A). First, we demonstrate that the general classification approach is able to decode information content from the pattern of activity observed after the cue presentation. This time-resolved pattern analysis demonstrates significant coding of the cue at around 100 ms (Figure 3B), corresponding to the time of rapid divergence observed in the distance metric (Figure 2B). Pattern classification also peaks at around 230 ms and remains relatively uniform into the delay period.

To directly assess the time stability of the activity state differentiating trial types, we decoupled the temporal windows used for train and test (see schematic in Figure 3A; see also Crowe et al., 2010; Meyers et al., 2008). If accurate generalization is observed across time (train at time t, test at time t+*n*), we can infer that the population code that differentiates trial type at time t is significantly similar to the coding scheme at time t+*n*. At the extreme, if the coding schemes were completely time stable, pattern classification should not be sensitive to which time points are used for test or train—by definition a stationary code does not vary across time. Conversely, if classifiers trained at time t are unable to decode patterns observed at time t+*n*, then we can conclude that population coding is time specific.

**Figure 4:**
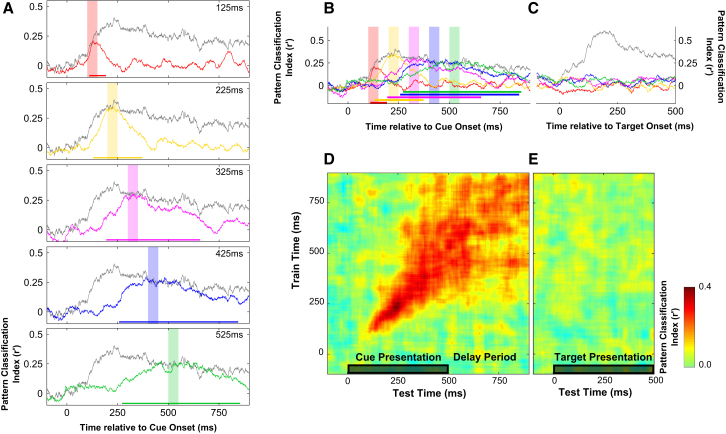
Cross-Temporal Pattern Analysis (A) Cross-temporal pattern classifiers were trained to discriminate trial type using data from the shaded 50 ms time window and tested throughout the cue duration and subsequent delay period. For reference, within-time classification performance is shown in gray to illustrate the upper limit of trial type information at each time point and significant periods of above-chance cross-temporal classification are indicated by the color-coded significance bar below the corresponding trace. The complete time course of these cross-temporal analyses is available online as Movie S2. (B) Cross-temporal classification results from (A) overlaid. (C) Cross-generalization was extended to the target epoch to test for prospective target coding following same conventions as (B), with gray trace reflecting the within-time classification for target type. (D and E) Full cross-temporal classification matrix. Classifiers are trained to discriminate trial type at each 50 ms time window (1 ms increments) during the cue and first delay period, and trial type discrimination is tested throughout the cue (D), first delay, and target period (E).

Cross-temporal classification results for trial type are presented in Figure 4. Different color traces represent classification performance for classifiers trained on data from corresponding shaded time windows and tested throughout the cue and delay epochs. For reference, the within-time classification performance is shown in gray in each plot to illustrate the envelope of trial type coding in the population response, i.e., the maximal trial type information at each time point.

Evidence for time-specific coding is most apparent at the onset of cue processing (see Figure 4A). Classifiers trained on the population response at 100–150 ms postcue onset (in red) only successfully discriminate between trial types for test data taken from proximal time windows: 100 ms to 200 ms. Classification is no better than chance at discriminating trial type from data taken at later times. This failure cannot be attributed to any lack of discriminative information at these subsequent time points after cue onset. On the contrary, within-time classification actually peaks at around 200 ms and is relatively sustained thereafter (shown in gray, Figures 4A). Therefore, the specific pattern of activity that differentiates condition between 100–150 ms is unique to this early stage of cue processing and does not persist beyond 200 ms or into the delay period.

Temporal specificity is also evident at the next training window, 200–250 ms. Again, pattern classification is optimal for data taken from the equivalent time period, relative to other time points, although there is a broader window of above-chance classification (at least 150–300 ms). This implies an increasing degree of time stability; however, cross-generalization still returns to chance levels before the offset of the cue stimulus. There is more evidence for time stability at 300 ms, and by 400–450 ms, there is clear evidence for stable coding into the delay period. This profile of increasing time stability accords with the reduction in multidimensional velocity observed toward the end of the cue onset period and into the memory delay period (Figure 2E).

Since the pattern of activity that drives robust classification during cue processing does not persist into the delay period, coding during the delay is unlikely to reflect passive persistence in firing. To test whether delay activity reflects prospective coding for the target stimulus (Rainer et al., 1999), we extended the cross-temporal analysis to the presentation of the target (Figure 4C). Again, the gray trace in Figure 4C illustrates the envelope of significant target-related information that was decodable using within-time pattern classification, i.e., train and test at equivalent time points after target onset. All other traces reflect the accuracy of target classification using the neural patterns observed during the color-coded windows in the cue period. At no stage does the pattern from cue and delay periods reflect the population response observed during any time of target processing, even though the population response contains significant target-discriminating information, as shown by the gray trace.

The full cross-temporal classification analysis is shown in Figures 4D and 4E. The diagonal axis through this matrix is identical to the within-time classification of trial type shown in gray in Figures 4B and 4C. Figure 4D clearly demonstrates the time specificity of population coding during the cue processing period, whereas population coding in the delay period is more time stable. Again, there is no evidence for cross-generalization of coding during the cue or associated delay period to the target-related response (Figure 4E).

The results so far suggest that information concerning trial type is maintained through the delay period as a stable low-energy state. Although the population response differentiates between the three alternative contexts, the underlying code does not resemble patterns observed during cue processing or the expected target. This nonstationary coding scheme contrasts with classic models of WM that posit persistent maintenance of the initial input representations (Miller et al., 1996; Wang, 2001) or preactivation of the expected target/memory probe (Rainer et al., 1999). We suggest that the postcue state could reflect a temporary reconfiguration of the tuning profile in prefrontal cortex for flexible behavior, i.e., to discriminate choice stimuli according to context for “go”/“no-go” decision making.

### Classification Driven by the Neutral Stimulus: Differential Patterns Elicited by Fixed Input

**Figure 5:**
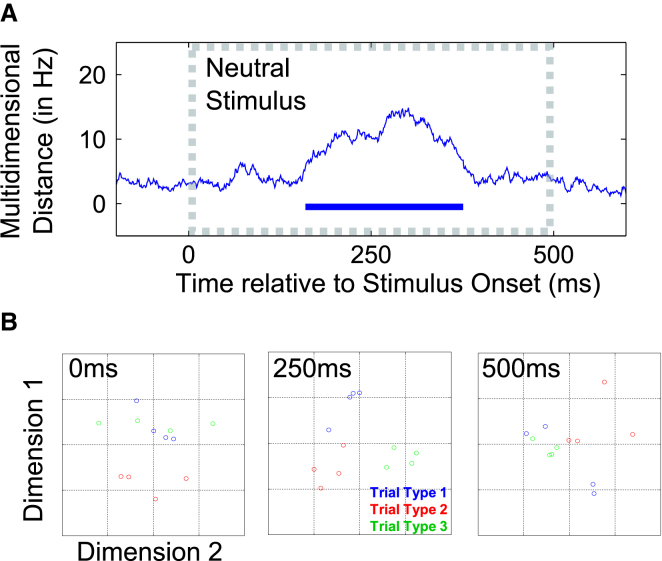
Trial Type Coding in Patterns Driven by a Fixed Neutral Stimulus (A) Multidimensional distance between the network responses to neutral stimuli presented within each trial type is plotted as a function of time, and the blue bar along the x axis indicates periods of above-chance classification. (B) The multidimensional distance is visualized using the first two dimensions defined by MDS. Each data point reflects an independent sample of the population response to neutral stimuli presented within each trial type (color coding as in Figure 1D). The complete time course of the temporary emergence of condition-specific clustering in state space is available online as Movie S3.

A systematic reconfiguration of the network state in prefrontal cortex would also be expected to alter the response characteristic of the network to fixed input (Mongillo et al., 2008; Sugase-Miyamoto et al., 2008). Indeed, we find that the population response to the neutral stimulus clearly differed as a function of trial type (Figure 5A), even though the same neutral stimulus was used for all trial types (see Experimental Procedures). This suggests that the activation profile of the network is patterned according to trial type. To visualize the separation of activity states driven by the fixed neutral stimulus, we plot four independent estimates of the activity pattern associated with each trial type (color coded) onto the first two dimensions determined by MDS (Figure 5B; the full time course is captured in the Movie S3 available online). Data points clearly cluster as a function of trial type at 250 ms after stimulus onset, reflecting systematic activity states that differentiate the response to the fixed neutral stimulus according to context.

### Cue-Dependent Processing of Choice Stimuli

We propose that cue processing establishes a state in PFC that temporarily tunes prefrontal neurons to respond according to the current task context, i.e., to decide the appropriate behavioral response to choice stimuli. In a final set of analyses, we examined responses to the three choice stimuli that, according to the rule established by the current cue, could serve as either a “go” or “no-go” signal for the behavioral response.

We defined stimulus 1 as the stimulus serving as a target with cue 1, but a distractor with cues 2 or 3, and similarly for stimulus 2 (target with cue 2) and stimulus 3 (target with cue 3). First, to track stimulus-driven coding, we trained the pattern classifier to discriminate between choice stimuli on trials in which they served as distractors (e.g., distractor 1 versus distractor 2) and tested classification performance on trials in which the same stimuli served as targets (e.g., target 1 versus target 2) within the corresponding time window. This procedure was performed for each possible pairing, and the results were averaged to calculate an overall classification score for stimulus-specific coding. Importantly, this cross-condition analysis tests specifically for context-independent coding of the physical properties of the choice stimuli. Only the pattern difference between stimulus types that is evident in both targets and distractors can contribute to decoding.

We also explored the stimulus-independent coding of behavioral category. For each of the stimuli 1–3, we trained classifiers to discriminate behavioral category (e.g., target 1 versus distractor 1) and tested performance on category discrimination of a different stimulus (e.g., target 2 versus distractor 2) within corresponding time windows. Again, the multiple pairwise tests were averaged to derive a single index of stimulus-invariant coding for the behavioral category: target versus distractor.

**Figure 6:**
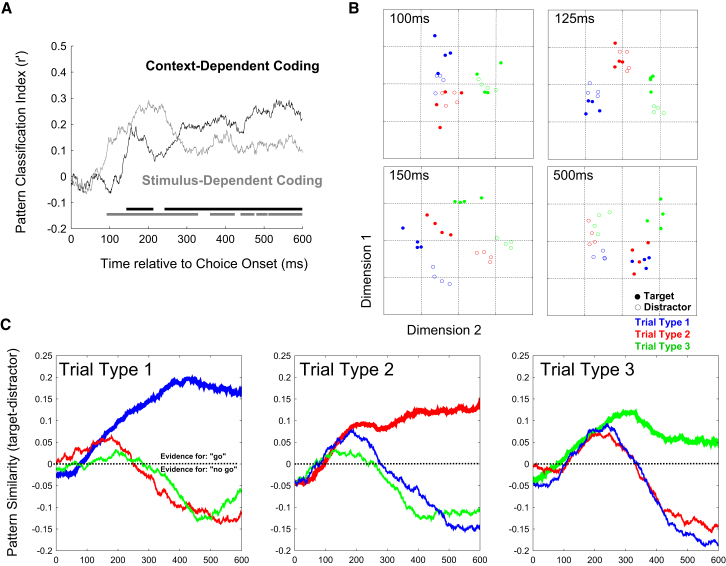
Evolution of Coding during Choice Processing (A) Cross-generalization pattern analysis reveals the time course of stimulus-dependent coding (in gray) and context-dependent coding (in black). Significant periods of above-chance discrimination are indicated by corresponding significance bars along the x axis. (B) State space is represented by the first two dimensions from MDS. Data points correspond to four independent estimates of the population response to each of the choice stimuli presented as a target (filled) or distractor (unfilled). Blue, stimulus serving as target with cue 1; red, stimulus serving as target with cue 2; green, stimulus serving as target with cue 3. The full time course of the coding transformation in state space is available online as Movie S4. (C) Evidence for a “go” or “no-go” decision to choice stimuli is plotted as a function of time after the presentation of each stimulus type within each trial type (separate plots). Color coding for the three stimuli are as in (B). Choice-related evidence is traced in heavy lines for “go” stimuli (i.e., targets) and in a thin line for “no-go” stimuli (i.e., distractors). Evidence for each choice is quantified relative to an independent reference pattern that differentiates go/no-go stimuli at the end of the trial: target minus distractor. Consequently, positive values reflect positive evidence for a “go” decision, whereas negative values reflect positive evidence for a “no-go” decision. Also see Figure S1 in online Supplemental Information.

The results in Figure 6A reveal the transition in PFC from stimulus-dependent to context-dependent coding. Initially, the population response discriminates between the physical properties of the different stimuli (from ∼90 ms, gray trace), but shortly afterward, stimulus-invariant coding for task-relevance also emerges in the pattern of activity (from ∼140 ms, black trace). This transition from stimulus-specific to context-dependent coding corresponds in time to a transient increase in the overall activity of the network. As the network again begins to settle toward a low-energy state (see Figure 1D), pattern differentiation is dominated by the choice decision (see Figure 6A).

The transition from stimulus-specific coding to context-dependent coding for choice events can also be visualized in the first two dimensions derived through MDS (Figure 6B). Data points correspond to four independent estimates of the multidimensional response to the three choice stimuli (color coded) presented as a target (filled circles) or distractor (unfilled circles). The first coherent organization in state space is observed around 100 to 125 ms and separates the response as a function of stimulus identity. There is very little separation by decision value (i.e., behavioral choice). Separation according to both parameters is evident by 150 ms, but by the end of the trial, the state space is most clearly differentiated by behavioral choice.

To explore in more detail how evidence for the choice-related response evolves in PFC, we track the evolution of the pattern match between the population response and either decision state (“go” versus “no-go”). Results are plotted as a function of stimulus (color coded) separately for each trial type (Figure 6C). The reference pattern for differentiating decision values was determined for each trial type by contrasting target and distractor population response observed at the end of the trial (i.e., 350–550 ms) in the other two trial types. Consequently, the reference pattern was statistically independent from the test data. Moreover, this cross-comparison approach also ensures that the decision-related coding scheme is shared across trial types. As such, accurate readout is determined according to a reference pattern that is effectively invariant with respect to time and trial type. Although this is a conservative estimate of behaviorally relevant coding, this level of abstraction would be ideal for robust decision making across contexts. The same readout strategy can be used irrespective of time or condition.

Finally, additional analyses presented in Supplemental Information show that positive evidence is accumulated for both decision values (i.e., some neurons are more active for “go” decision relative to “no-go,” whereas others show the opposite pattern; see Figures S1A and S1B; see also Kusunoki et al., 2010). Moreover, we found no evidence that the eventual decision state was represented by a distinct population of neurons functionally distinguishable from early stimulus-selective cells (see Figure S1C). Rather, the final decision state appears to emerge within the same functional network that initially codes the physical properties of the choice stimuli.

## Discussion

In this study, we use dynamic pattern analysis to characterize how prefrontal cortex establishes, maintains, and uses flexible cognitive states for task-dependent decision making. Population-level analyses demonstrate how an instruction cue triggers a complex trajectory through state space, beginning with a rapid sequence of highly reliable cue- and time-specific patterns during the most active phase of the evoked response. After these high-energy state transitions, the population returned to a less active stable state that persisted throughout the first delay period. Although activity patterns in the delay period were context specific, coding of trial type in this relatively quiescent state did not resemble the representational structure of previous cue or anticipated choice stimuli. Thus, we find no evidence that cue-related activity persists as an active representation in WM or that delay activity reflects preactivation of the target stimulus. Rather, we argue that delay activity reflects a distinct neurophysiological state established during cue processing. This context-dependent state temporarily sets the tuning profile of PFC according to the current task demands, i.e., to classify each subsequent choice stimulus as either a go or no-go response signal.

**Figure 7:**
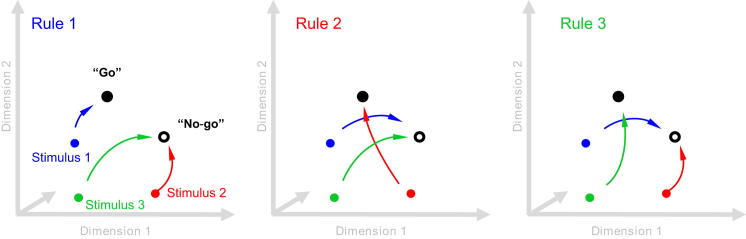
A Simple Schematic of the Proposed Tuning Mechanism Depending on the context, input matching a particular choice stimulus is routed along a context-dependent trajectory toward an activity state that codes behavioral choice.

Consistent with this proposal, we found that coding in PFC during the decision-making process initially reflected the physical properties of the choice stimulus before differentiating between the two alternative decision values: “go” versus “no-go” (Figure 6). In separate analyses, we also demonstrate that positive evidence for both these decision values contributes to the choice-discriminating coding scheme (Figures S1A and S1B). We also find no clear functional delineation between neurons coding the stimulus properties during the earliest processing phase and the neurons that ultimately code for the behavioral choice (Figure S1C). Analyses of choice processing thus demonstrates how tuning in this population of prefrontal cells is determined by task context. This distinct state determines a trajectory through activity space that effectively maps distinct stimuli to the appropriate decision value according to context (see schematic in Figure 7).

### Representing Behavioral Context

To solve the sequential demands of this task, information about trial type needs to be maintained across delays and interference to inform decision making at each choice stimulus. Prefrontal cortex has long been associated with distractor-resistant maintenance in WM (Miller et al., 1996) via persistent firing of stimulus-specific neurons (Wang, 2001). Possibly, therefore, the temporal gap in this task might be bridged by an active WM representation, allowing decision making to operate directly on two sources of information: memory representation of the cue and perceptual representation of the choice stimulus. However, we find that the cue triggers a series of time-specific activity states rather than a persistent static state. Although activity does eventually stabilize during the delay period, the coding scheme is effectively orthogonal to coding driven by the cue stimulus. Cross-temporal pattern analysis has previously identified similar dissociations between the stimulus-driven response and subsequent memory-related delay activity in prefrontal and parietal cortex across a range of tasks (Barak et al., 2010; Crowe et al., 2010; Meyers et al., 2008).

This task could also be solved by selectively preactivating the target-related pattern in response to the cue and in anticipation of the choice stimulus (Rainer et al., 1999). The behavioral decision could then be made according to the match (or mismatch) between the internal target representation and the sensory input. Preactivation of a target representation has often been proposed as a critical aspect of attentional control, for example, in biasing attentional competition (Desimone and Duncan, 1995), and preactivation in visual cortex has been described in both human (Stokes et al., 2009) and monkey (Chelazzi et al., 1998). In our case, however, PFC did not engage similar mechanisms.

Although we find no evidence that delay activity resembles target-related coding (Figure 4), our data are not inconsistent with previous evidence that preparatory activity in PFC reflects target expectation (Rainer et al., 1999). Using a paired-associate WM task, Rainer et al. (1999) found that delay activity was more selective for the anticipated stimulus than the memory stimulus. However, selectivity was assessed within isolated time windows, so it was not possible to test whether prospective coding reflected preactivation of the target representations per se. Using a variant of their paired-associate task, we now extend their key findings to show that selectivity is unique to the delay period. Our data are still broadly consistent with a prospective coding model, insofar as the memory state is configured for future task demands, but we suggest that prospective coding is not implemented through preactivation of a sensory target representation.

Our results may also be compared with those from the oculomotor delayed response task (e.g., Takeda and Funahashi, 2004), in which an initial cue (a stimulus in the peripheral visual field) is followed after a delay by a saccade to the cued location. In this case, the strong tendency is for prefrontal neurons to have matched spatial preferences across cue, delay, and response epochs (Takeda and Funahashi, 2004). If the response is to be made to a location that is different from the initial stimulus location, then spatial vectors of population activity rotate through the trial period from an initial coding of stimulus location to a final coding of response position, again presuming fixed spatial preference in individual cells. Importantly, in the oculomotor delayed response task, response preparation can begin at the time of initial stimulus presentation, unlike the case in cued paired-associate or delayed matching tasks. When a cue instructs an arbitrary rule for classification of subsequent stimuli, our data show that patterns of cue, delay, and target coding can be entirely independent.

### Context-Dependent Decision Making

Analysis of choice processing demonstrates an early stimulus-driven response pattern, which is rapidly transformed into a more stable choice-related coding scheme (Figures 6A and 6B). Effectively, the context provided by each trial type allows context-independent stimulus coding to be transformed into a stable state coding for the appropriate behavioral response (Figure 6C). Interestingly, choice stimuli appear to drive positive evidence for both decision values (Figure S1; see also Kusunoki et al., 2010). This is more consistent with adaptive routing of processing trajectories for context-dependent decision making (Figure 7) than an attentional gate to filter out task-irrelevant stimuli. In this task, both “go” and “no-go” signal signals are important for correct behavior; the challenge, therefore, is to discriminate between these signals, rather than simply to detect the target stimulus. Attentional gating might be more important if competing stimuli are presented simultaneously (e.g., Chelazzi et al., 1998).

Finally, we also found that transient stimulus-specific coding during the initial response to choice stimuli was distributed within the same neural population that later settles into the more stable decision state (Figure S1). This suggests that the trajectory through the state space remains effectively within the same neural population (see also Jun et al., 2010), presumably via a complex web of local interconnections. Decision making in this region of prefrontal cortex is therefore best characterized as a transition from a context-invariant state to context-dependent coding within the same functional network.

### Dynamic Population Coding

Our results are consistent with an adaptive coding model of prefrontal cortex in which flexible goal-oriented behavior is mediated via dynamic changes to prefrontal tuning properties (Duncan, 2001; Duncan and Miller, 2002). As in previous studies (e.g., Freedman et al., 2001; Meyers et al., 2012; Watanabe, 1986), we show that PFC processes input as a function of task relevance. Here we provide a detailed picture of the underlying network dynamics, from rule encoding and maintenance to context-dependent decision making.

A plausible mechanism for flexible tuning is activity-dependent short-term synaptic plasticity (Zucker and Regehr, 2002). Short-term plasticity has recently been identified as a possible basis for maintaining information in WM (Erickson et al., 2010; Fujisawa et al., 2008; Mongillo et al., 2008). If patterned activity leaves behind a patterned change in the synaptic weights of the network (i.e., hidden state), then subsequent stimulation will be patterned according to the recent stimulation history of a network (Buonomano and Maass, 2009). Thus, any driving input to the system will trigger a systematic population response that could be used to decode the recent stimulation history of the network (Nikolić et al., 2009). Exactly this phenomenon is seen in our data during the presentation of the neutral stimulus (Figure 5). Although this stimulus was fixed across trials, the population response was patterned according to the identity of the previous cue, providing a more reliable readout of the memory content than the population response observed during the relatively quiescent delay period. Recent WM studies in human (Lewis-Peacock et al., 2012) and nonhuman primate (Barak et al., 2010) have also proposed a similar mechanism for maintaining the contents of WM.

Short-term synaptic dynamics could also explain nonstationary population activity profiles, as observed here (Figure 4) and in other studies (Barak et al., 2010; Crowe et al., 2010; Meyers et al., 2008). If the hidden state of the network is continually altered by each pattern of activity, then even constant input to the system should result in time-varying patterns (Buonomano and Maass, 2009). Indeed, it could be relatively difficult to engineer a network that maintains a static activity state in the presence of activity-dependent short-term plasticity.

Finally, adaptive changes in tuning mediated by short-term synaptic dynamics could also explain the differential activity states observed during the delay period. Analysis of the neutral stimulus suggests that differences in the underlying hidden state can be revealed by increasing overall activity in the network. Following the same logic, baseline spontaneous firing within the network could also be sufficient to emit a decodable response pattern (Sugase-Miyamoto et al., 2008). In this respect, delay activity could be thought of as the response function of the network, rather than active maintenance of a static firing state.

Here, we highlight short-term synaptic dynamics as an attractive putative mechanism for rapid adaptive coding in PFC (see also Buzsáki, 2010; Deco et al., 2010). However, other phenomena that systematically shift the response properties of a population could also contribute to adaptive coding. For example, temporary activity-dependent changes in membrane potentials could also shift the tuning profile of the network (Buonomano and Maass, 2009). Moreover, a systematic shift in the baseline activity state of the network could reroute processing via conditional logic gates (McCulloch and Pitts, 1943) and/or exploiting nonlinear dynamics (Izhikevich, 2007). Finally, neural synchrony might be especially important for temporary shifts in effective connectivity (Fries, 2009). Phase synchrony has been implicated in WM (Axmacher et al., 2010; Buschman et al., 2011; Fell and Axmacher, 2011), and a recent study has further shown how rapid configuration of synchronized networks in PFC is specific to different rules states (Buschman et al., 2012). These mechanisms might also be able to implement the functional change we describe here—a context-dependent shift in network dynamics, altering the mapping of sensory inputs to final behavioral decisions.

## Experimental Procedures

### Subjects

Subjects were two male rhesus monkeys (Macaca mulatta), weighing 11 and 12 kg. All experimental procedures were approved by the UK Home Office and were in compliance with the guidelines of the European Community for the care and use of laboratory animals (EUVD, European Union directive 86/609/EEC).

### Task and Stimuli

The cued target detection task is schematized in Figure 1B. Each trial commenced with a 500 ms baseline period, during which the monkey held fixation on a red central fixation point accompanied by two dim gray circles (location markers) 6° to left and right on the horizontal meridian. Next, one of three cue stimuli was presented for 500 ms at either the left or right (randomized) location marker. The cue determined the spatial location of all subsequent stimuli within that trial and also the direction of the eventual saccade response at the end of the trial. Most importantly, the cue identity also instructed which choice stimulus would be the target stimulus for the current trial. During initial training sessions, monkeys learned to associate three specific cue stimuli with three specific target stimuli. An additional stimulus served as a neutral nontarget item. All pictures were randomly drawn from the same set of images (2° × 2°). New stimulus pairs and neutral pictures were occasionally introduced and maintained for a number of sessions. Data were included only after at least six training sessions with a given stimulus set, at which time this set was highly familiar and performance had reached asymptote (see Kusunoki et al., 2009).

After cue presentation, between zero and three nontarget stimuli were presented at the same location as the cue and finally the cue-associated target. Each stimulus was presented for 500 ms, with a random delay of 400–800 ms between each stimulus and the next. Nontarget stimuli were randomly drawn with replacement from the set of two stimuli serving as targets on other trials (“distractors”) and the neutral stimulus. Target probability remained constant at 0.3 for the first three sequential positions after the cue. If three nontargets had been presented, target probability increased to 1.0, thus obviating the need for cue-specific stimulus categorization. Consequently, responses to targets presented after three nontargets were not analyzed. At target offset, monkeys were required to make a saccade to the location placeholder on the side of stimulus presentation.

Correct performance (accurate saccade with latency <500 ms) was rewarded with a drop of juice. The trial was immediately terminated after any other break from fixation. The window size for both central fixation and end point of saccade to target location was ≤3.5° × 3.5° for 78.4% of the recorded cells and 5° × 7° (fixation) and 5° × 5° (target location) for the remaining cells.

### Recordings

Each monkey was implanted with a custom-designed titanium head holder and recording chamber (Max Planck Institute), fixed on the skull with stainless steel screws. Chambers were placed over the lateral PFC of the right hemisphere for monkey A at anterior-posterior = 32.0, mediolateral = 22.2, and the left hemisphere for monkey B at anterior-posterior = 25.8, mediolateral = 21.2. Recording locations for each animal are shown in Figure 1C, which included BA 8, 9/46, and 45.When task training was completed, a craniotomy was made for physiological recording. All surgical procedures were aseptic and carried out under general anesthesia. We used arrays of tungsten microelectrodes (FHC) mounted on a grid (Crist Instrument) with 1 mm spacing between adjacent locations inside the recording chamber. The electrodes were independently controlled by a hydraulic, digitally controlled microdrive (Multidrive 8 Channel System; FHC).

Neural activity was amplified, filtered, and stored for offline cluster separation and analysis with the Plexon MAP system (Plexon). Eye position was sampled at 100 Hz using an infrared eye tracking system (Iscan) and stored for offline analysis. We did not preselect neurons for task-related responses; instead, we advanced microelectrodes until we could isolate neuronal activity before starting the search tasks. Data were obtained from a total sample of 627 cells. At the end of the experiments, animals were deeply anesthetized with barbiturate and then perfused through the heart with heparinized saline followed by 10% formaldehyde in saline. The brains were removed for histology, and recording locations were confirmed on dorsal and ventral frontal convexities and within the principal sulcus.

### Data and Analysis

Physiological data were analyzed from only successfully completed trials, on average including 50 repetitions for each combination of trial type (cues 1–3) and choice stimulus. All statistical analyses were performed using MATLAB (MathWorks).

### Mean Activity

Unless otherwise specified, the instantaneous firing rate was estimated by the mean spike count within a 50 ms sliding window. The overall population response to each stimulus type was assessed relative to the prestimulus baseline period (−100 to 0 ms) with univariate statistical analyses.

### Network Dynamics

The activation state of the full population was represented as a 627-dimensional coordinate in Euclidean space, where each dimension represents the instantaneous firing rate of a single neuron estimated within 50 ms sliding windows. The dynamic trajectory through this state space is the path that passes through the multidimensional coordinate of each time point.

To explore the dynamic behavior of this network, we first calculated the Euclidean distance between trajectory pairs (e.g., D(Cue1,Cue2)) as a function of time. Distances between all contributing pairs of conditions (e.g., all pairs of cues) were then averaged to yield a single summary statistic for each time point. Randomized permutation tests were performed using exactly the same approach but with randomized condition labels for each sample. Because distance is always positive, distance values were expressed relative to the median of the permutation test, and statistical significance was inferred relative to the observed null distribution. This distance measure is closely related to the population coding metric described below—accurate pattern decoding critically depends on a reliable multidimensional distance between conditions. Position in state space was visualized using classical MDS. For each analysis, we made four independent estimates of the population activity for each condition of interest by averaging every fourth trial within that condition. MDS was then performed on the Euclidean distance matrix. Data were plotted against the first two dimensions. The distribution of the four within-condition data points provides a sense of the tightness of condition-dependent clustering.

We also consider the speed of activity trajectories through state space. In general, the instantaneous velocity at time t can be estimated by calculating the difference in activity state as a function of time: d(P1_t-*n*_, P1_t+*n*_)/2*n*. Here, we used n = 20 ms. As in all other analyses, firing rate was estimated within a 50 ms sliding window. This metric is equivalent to summing the absolute slope of each firing rate (see Kusunoki et al., 2009) and is sensitive to mean changes in the overall activation level of the system. However, most importantly, this metric is also able to identify changes in patterns that are not associated with a mean change in energy. For comparison, we can also consider the change in mean firing rate across neurons, i.e., the overall change in the energy level of the system.

### Population Decoding

Pattern analysis was also performed on the full population vector (n = 627) of instantaneous firing rates estimated within 50 ms sliding windows for each condition of interest. For each pairwise test (e.g., cue 1 versus cue 2; cue 1 versus cue 3; cue 2 versus cue 3), we first subdivided the samples into train and test data sets using an interleaved approach (e.g., averaging across odd and even cue 1 and cue 2 trials). Using an interleaved subdivision of the data reduces extraneous differences between train/test subdivisions caused by drift in the neural response across the testing session. Next, we contrasted the activity profiles between the conditions of interest to derive two independent estimates of the condition discriminative pattern across neurons, e.g., Train_Cue1-Cue2_; Test_Cue1-Cue2_. Finally, the pattern similarity between these differential population vectors was quantified by a Fisher-transformed Pearson correlation, *r′*. A positive correlation coefficient indicates reliability across the independent data sets and thus evidence for a reproducible condition-specific difference across the neural population. For multiclass decoding (e.g., cue 1 versus cue 2 versus cue 3), we repeated this for each pairwise combination and used the mean correlation coefficient as the overall summary statistic. Statistical significance was assessed using randomized permutation testing (see below).

To establish the temporal evolution of information coding in PFC, we first applied pattern analysis by training and testing classifiers on data from equivalent time points. This analysis is conceptually very similar to the multidimensional distance metric described above but using a measure of similarity to test the generalizability of condition-specific patterns, rather than a measure of dissimilarity to quantify the absolute difference between activity vectors.

Importantly, the classification approach can be easily extended to test for cross-generalization over different time points. In this cross-temporal extension, we train and test at different equivalent time points. Above-chance cross-temporal generalization provides evidence for a time-stable population code, whereas a failure to generalize across time suggests that coding is time specific.

The cross-generalization approach is also easily extended to test for similarity between coding schemes. For example, we also trained our pattern classifier on differences between the physical identities of two choice stimuli on trials in which they were targets (e.g., target 1 versus target 2) and tested on trials in which the same stimuli were distractors (e.g., distractor 1 versus distractor 2). This provides a formal measure of the shared pattern between the two contexts.

### Statistical Analyses

We used standard parametric univariate statistics to examine the overall mean firing rate. All other statistical significance testing was performed using assumption-free randomized permutation testing. For the multidimensional distance analyses, the null distribution was estimated from 1,000 permutations of randomly shuffled condition labels using exactly the same procedure as the main test. The median of the distribution of randomized distances was then subtracted from the observed distance between conditions, and the 95% confidence intervals were used to determine the threshold for detecting a significant difference from chance (i.e., p < 0.05, two-tailed). To control for multiple comparisons in the time course analyses, we also estimated the distribution of the number of contiguous above-threshold classifications expected by chance. Only temporal clusters exceeding the 95% cutoff threshold were presented in each plot. Exactly the same procedure was performed for the classification-based pattern analyses.

## Supplemental Information

Document S1. Figure S1 and Supplemental Experimental Procedures

Movie S1. Cue MDS The full time course of cue-related coding in prefrontal cortex as visualized with the first two dimensions derived by multidimensional scaling (MDS). This dynamic series accompanies the selected frames shown in Figure 2D.

Movie S2. Cross-Temporal Cue Classification The full time course of time-specific cue-related coding in prefrontal cortex revealed by cross-temporal pattern analysis, as shown in Figure 4.

Movie S3. Neutral MDS The full time course of trial type discrimination during the presentation of the neutral stimulus as visualized with the first two dimensions derived by MDS. This dynamic series accompanies the selected frames shown in Figure 5B.

Movie S4. Choice MDS The full time course of trial type, and stimulus type, discrimination during choice processing as visualized with the first two dimensions derived by MDS. This dynamic series accompanies the selected frames shown in Figure 6B.
