# Supplementary material for: Dynamic Coding for Cognitive Control in Prefrontal Cortex
Source: Neuron. 2013 Apr 24;78(2):364–75. doi: 10.1016/j.neuron.2013.01.039 (PMC3898895; doi:10.1016/j.neuron.2013.01.039)
Supplement: Document S1. Figure S1 and Supplemental Experimental Procedures [file mmc1.pdf]

Neuron, Volume *78*

Supplemental Information

Dynamic Coding for Cognitive Control in Prefrontal Cortex

Mark G. Stokes, Makoto Kusunoki, Natasha Sigala, Hamed Nili, David Gaffan, and John Duncan

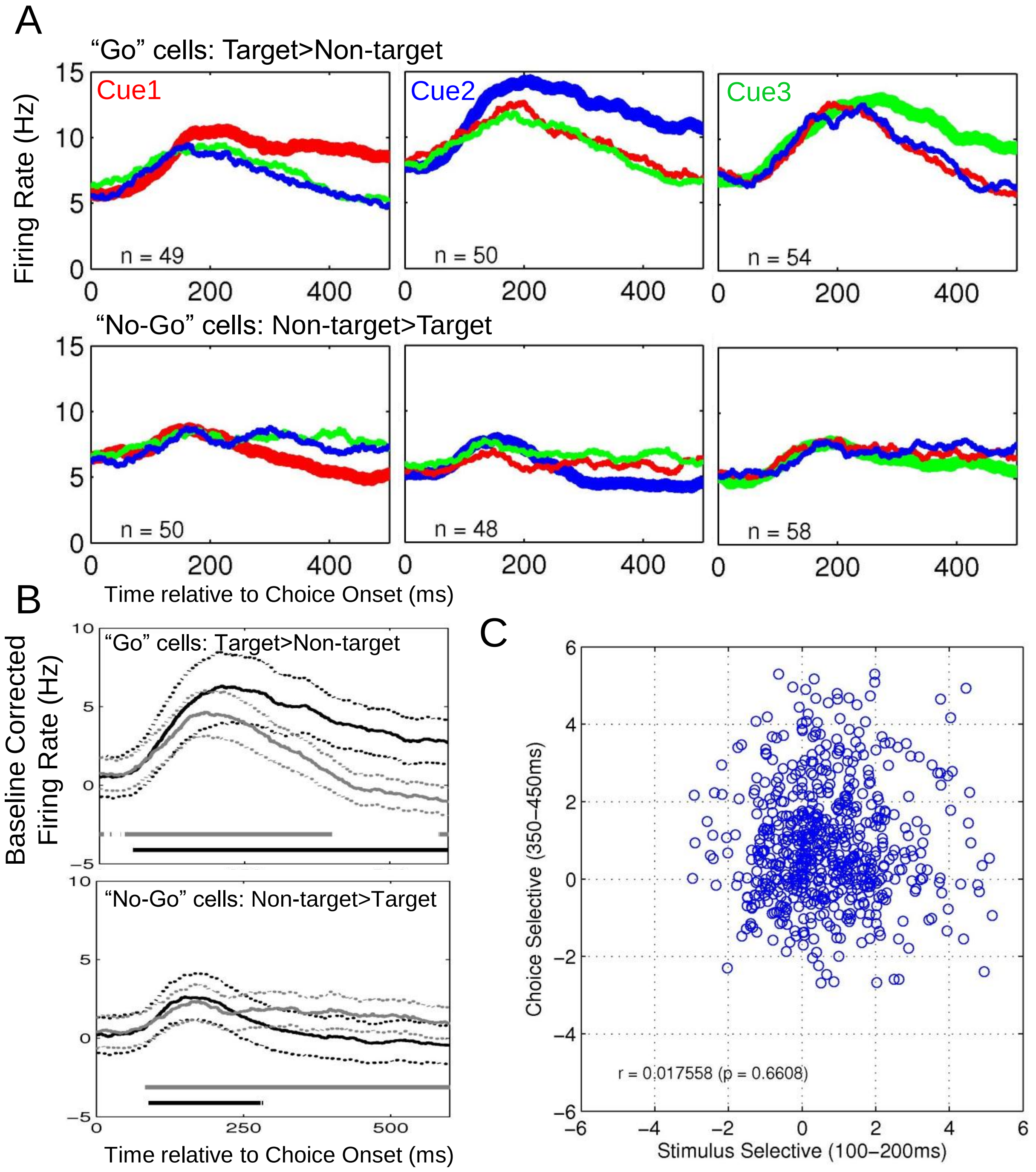
A
"Go" cells: Target>Non-target
Cue1
Cue2
Cue3
Firing Rate (Hz)
n = 49
n = 50
n = 54
"No-Go" cells: Non-target>Target
n = 50
n = 48
n = 58
Time relative to Choice Onset (ms)
B
Baseline Corrected Firing Rate (Hz)
"Go" cells: Target>Non-target
"No-Go" cells: Non-target>Target
Time relative to Choice Onset (ms)
C
Choice Selective (350–450ms)
Stimulus Selective (100–200ms)
r = 0.017558 (p = 0.6608)

Figure S1. Choice related activation of a subset of "go" versus "no-go". (**A**) Cells were classified as "go" (upper panel) of "no-go" cells (lower panel) based on an independent estimate of firing rate at 400-500ms after the onset of the choice stimulus. Activity in these independently selected subpopulations is shown as a function of choice stimulus (colour coded), and plotted separately for each cue context. Choice processing is characterised by a selective activity increase in "go" and "no-go" cells. (**B**) The same data as (A), but averaged over cue and stimulus. Broken lines reflect $CI_{95\%}$, and significant periods of above-chance discrimination are indicated by corresponding significance bars along the x-axis. (**C**) Scatter plot to test for the relationship between early (100-200ms) stimulus-selective coding and later (350-450ms) choice selective coding.

SUPPLEMENTAL EXPERIMENTAL PROCEDURES

We tested the relative contribution of evidence for both decision values explicitly by tracking the time-course of activity in “go” and “no-go” decision neurons during choice processing. The decision value for each neuron was evaluated within an independent context, thus avoiding circularity in our analysis, but also ensuring that the decision states of interest generalize across the different cues (i.e., not idiosyncratic to the stimulus type or cue condition). The firing rate for “go” (upper panel) and “no-go” decision cells (lower panel) is plotted as a function of choice stimulus (colour coded) separately for each cue context (**Figure S1A**) and pooled across cue type (**Figure S1B**). This analysis is closely related to the classification approach described above (in **Figure 6**), but allows the contribution of both decision values to be assessed explicitly. After an early transient non-specific response, the cued choice stimulus selectively drives activity in “go” cells, whereas only the uncued choice stimulus drives the activity in “no-go” cells. This suggests that positive evidence for both decisions contribute to the behavioural choice. We also tested whether the evolution from stimulus-specific to choice-related coding is best characterised by a transition between distinct neural populations, or an evolution within the same population. Stimulus selectivity was estimated between 100-200ms after the choice stimulus and choice selectivity at 350-450ms. As shown in **Figure S1C**, we found no relationship between the population engaged in early stimulus-selective processing and later choice selectivity (r=.01, *p*=.66).
